# Caspase-1-mediated cytokine release from gestational tissues, placental, and cord blood

**DOI:** 10.3389/fphys.2015.00186

**Published:** 2015-06-23

**Authors:** Ebtehaj Maneta, Averil Y. Warren, Daniel P. Hay, Raheela N. Khan

**Affiliations:** Division of Medical Sciences and Graduate Entry Medicine, School of Medicine, The Royal Derby Hospital, University of NottinghamDerby, UK

**Keywords:** pregnancy, parturition, inflammasome, IL-1β, caspase-1, P2X_7_ receptor

## Abstract

Distinguishing between fetal and maternal inflammatory responses is necessary for understanding the immune interplay either side of the placenta. Fetal immunity reaches maturity during extrauterine life and while basic inflammatory responses afford a certain degree of protection, fetuses are vulnerable to infection. With the discovery of inflammasomes—intracellular scaffolds that facilitate the elaboration of reactions resulting in the release of mature interleukin-1β (IL-1β)—it is necessary to consider how inflammatory stimuli are processed. The purinergic P2X_7_ receptor located on haematopoietic cells is a key intermediary in signal transduction initiated at Toll-like receptors (TLR) terminating in release of the mature IL-1β product. We demonstrate herein that IL-1β release from fetal membranes and mononuclear cells isolated from cord, placental, and maternal blood, obtained at term, is P2X_7_- and caspase-1 dependent. The P2X_7_-dependent release of the cytokine, which was highest from choriodecidua, was attenuated by progesterone (P4), prolactin and an NFkB inhibitor. The NLRP3 inflammasome appears necessary for the processing of IL-1β in gestational tissues and leukocytes.

## Introduction

Preterm birth (PTB) encompasses a spectrum of adverse pregnancy conditions with the majority being spontaneous in onset (sPTB) and linked to an aggravated inflammatory response (Goldenberg et al., 2008). Globally, over half of the 2.9 million neonatal deaths are attributed to infections and complications resulting from PTB (Lawn et al., 2014). Despite several proinflammatory cytokines being implicated in sPTB, targeting inflammatory pathways either as biomarkers for predicting the onset of sPTB and its progression or as potential therapeutic targets has proven difficult as a result of the complex immune interactions at the fetomaternal interface.

Inflammation may be elicited by danger-(damage)—or pathogen-associated molecular patterns, DAMPs, and PAMPs (Bianchi, 2007) respectively that activate downstream pattern recognition receptors (PRRs). PAMPs are typically microbial products (e.g., lipopolysaccharide, (LPS) bacterial/viral DNA) that signal through the family of membrane-bound Toll-like receptors (Takeda and Akira, 2005). In contrast, DAMPs although similarly diverse, include host-derived molecules such as ATP but also crystalline/particulate matter that may be of endogenous or exogenous origin. Many of the signaling cascades activated by PAMPs and DAMPs converge onto a multiprotein complex, the inflammasome which mediates a series of intracellular reactions terminating in the processing of the master proinflammatory cytokine interleukin (IL)-1β (Martinon et al., 2002; Tschopp et al., 2003) Inflammasome activation itself results from the assembly of individual proteins of which the NOD-like receptor (NLR) entity containing a pyrin domain (NLRP), ASC (apoptosis-associated speck-like protein with CARD domain) and pro-caspase-1 are in most cases, necessary components (Martinon et al., 2002; Tschopp et al., 2003). To date, four distinct types of inflammasomes, based on the central NLR have been characterized (Schroder and Tschopp, 2010) of which the NLRP3 inflammasome is of particular interest to us given its activation by an array of PAMPs and DAMPs implicated in PTB (Khan and Hay, 2014).

Unlike most cytokines, IL-1β is a leaderless protein, post-translational proteolytic cleavage of which is required for the release of its mature 17 kDa form (Perregaux and Gabel, 1994) and in monocytes occurs rapidly on inflammasomes. The latter in pregnancy has been little studied to date but some observations, for example, raised levels of the DAMP, high mobility group box-1, HMGB-1 (Romero et al., 2011) point tentatively to inflammasome involvement. Amniotic fluid caspase-1 levels have also been shown to be highest in women with sPTB with confirmed intrauterine infection compared with women throughout pregnancy without infection (Gotsch et al., 2008) while monosodium urate (a DAMP linked with the lysosmal damage pathway of NLRP3) causes increased IL-1β production in trophoblasts (Mulla et al., 2011).

The NLRP3 inflammasome is triggered in part by toxins including lipopolysaccharide (LPS) stimulation of TLRs (Mariathasan et al., 2006). Expression of TLR4 in the human placenta was first reported in trophoblasts covering peripheral chorionic villi (Holmlund et al., 2002). TLR4 immunoreactivity is increased in placentae of preterm cases with chorioamnionitis compared with those of preterm or term placentae without chorioamnionitis (Kim et al., 2004). However, in human myometrium, expression of TLR4 was unchanged in laboring samples (Youssef et al., 2009) in contrast to results from a mouse model showing a significant increase in TLR4 expression in laboring compared with non-laboring myometrium. Expression of TLR4 has also been reported in preterm and term placentae of normal women and those with chorioamnionitis (Kumazaki et al., 2004) and in myometrium and decidua (Krikun et al., 2007).

A well-characterized route linking TLRs with the NLRP3 inflammasome is via an ATP-gated purinergic receptor, P2X_7_ (Mariathasan et al., 2006) which we have previously shown to be expressed and functional in cord blood mononuclear cells (CBMCs) (Warren et al., 2008). While TLR4 leads to increased synthesis of mRNA for the precursor form of IL-1β, processing to its mature and releasable form is reliant upon the exit of K^+^ from cells—a role performed by the P2X_7_ receptor (Kahlenberg et al., 2005). Thus, while numerous studies have shown links between TLR4 and LPS to cytokine release, stimulation with ATP increases IL-1β release further through the P2X_7_-receptor and may be a missing ingredient in understanding sPTB. The aim of our study was to investigate whether key players in the inflammasome pathway are implicated in the release of IL-1β from mononuclear cells purified from maternal, placental, and cord blood obtained at term and in gestational tissues.

## Materials and methods

### Patient recruitment and sample collection

Ethical approval for the study was obtained from the Derbyshire Research Ethics Committee (Ref: 09/H0401/90). All myometrial, placental, and blood samples were obtained from patients attending the Department of Obstetrics and Gynaecology, Royal Derby Hospital. Patients provided informed, written consent prior to undergoing either a normal vaginal delivery or elective cesarean section (CS) at term gestation (> 37 weeks). Blood samples from normal deliveries were assigned as labor (L) while those from elective CS were designated non-labor (NL). Indications for elective cesarean section included maternal request, previous elective CS or breech presentation while cases with diabetes, hypertension, pre-eclampsia, were excluded. Placentae, once checked by the midwife and with the cord clamped, were transported to the lab within 20 min of delivery. Approximately 10–20 ml of cord blood were immediately harvested from the umbilical vein, dispensed into suitable aliquots into sterile heparinized tubes (BD vacutainer LH PST™ II) and mixed gently to prevent coagulation. For collection of placental blood (from the intervillous space), the placenta was gently squeezed, the pooled blood between the placental cotyledons collected and added to heparinized tubes. Maternal peripheral blood samples (10 ml) from the median cubital vein were taken by a registered midwife from pregnant women attending antenatal clinic and dispensed into tubes (BD vacutainer LH PST™ II) following transfer to the lab for processing.

### Isolation of mononuclear cells from blood samples

Mononuclear cells were isolated from cord blood (CBMC), placental blood (PLBMC) and maternal blood (MBMC) using Histopaque® density gradient medium. Ten ml of heparinized cord blood were diluted with 10 ml of serum free RPMI (RPMI 1640 medium + 5 mM L-glutamine (Invitrogen, Paisley, Scotland). Then, 10 ml of diluted blood was gently layered onto an equal volume of Histopaque®-1077 medium followed by centrifugation at 400 × g for 30 min at room temperature. After centrifugation, the opaque middle layer consisting of leukocytes was then transferred into a new tube, RPMI added (without serum) and the cell suspension centrifuged again at 250 × g for 10 min at room temperature. Following this step, 1 ml of red blood cell lysing buffer (Hybri-max® liquid, Sigma-Aldrich, Poole, UK) was added to the cell pellet and mixed for 1 min before 10 ml of RPMI was added followed by centrifugation at 250 × g for 10 min at room temperature. After aspirating the supernatant, the pellet was resuspended in 1 ml of RPMI, cell counts performed using a haemocytometer and viability of purified cell preparations assessed using trypan blue. MBMCs and PLBMCs were also isolated using this protocol.

### Stimulation and cytokine detection

Isolated CBMCs, PLBMCs, and MBMCs were plated at a density of 1–5 × 10^6^ cells/ml in 24 well plates and cells left to adhere overnight at 37°C in a CO_2_ incubator. On the following morning, culture media were aspirated prior to priming cells with 1 μg/ml^−1^ lipopolysaccharide (LPS) in RPMI for 4 h. The effects of a range of agonists and antagonists were tested by preincubating cells with the test chemical during LPS stimulation but prior to the addition of ATP. Thus, after 2 h of incubation with LPS, drugs were added to corresponding wells. Half an hour before the end of the 4 h total incubation period, 200 nM (3′-O-(4-benzoyl)benzoyl-adenosine 5′-triphosphate (BzATP) was added to each well to initiate IL-1β release. Controls consisted of unstimulated cells (without LPS + BzATP) maintained in culture media or with vehicle (dimethyl sulfoxide or ethanol). After incubations, supernatants were removed, centrifuged to remove cell debris, and aliquots frozen at −20°C until required for quantitation of IL-1β release by enzyme-linked immunosorbent assay (ELISA; Quantikine human IL-1β ELISA kit; R&D Systems, Abingdon, UK). ELISAs were performed according to the manufacturer's instructions. The sensitivity of the assay was <1 pg/ml^−1^, with an interassay variability of 4.9% (coefficient of variation) over 20 assays and does not distinguish between pro–IL-1β and IL-1β. The concentrations of LPS, progesterone (P4) and prolactin and time of incubation were determined empirically. The drugs tested were LPS (TLR4 agonist), BzATP (P2X_7_ receptor agonist), oATP: oxidized ATP (P2X_7_ inhibitor), KN-62 (non-competitive P2X_7_ antagonist), YVAD-CHO (caspase-1 inhibitor), 100 nM N4-[2-(4-phenoxyphenyl)ethyl]-4,6-quinazolinediamine (QNZ; NFkB inhibitor; Tobe et al., 2003).

### Flow cytometry

Primary antibodies (CD45, CD14, CD68; all from Abcam, Cambridge, UK) were added to 100 μl samples of CBMCs, PLBMC, and MBMCs into 4 ml EDTA tubes. Tubes were vortexed for 5 s then left in the dark for 30 min followed by the addition of FITC-labeled secondary antibodies. Cells were then fixed in 500 μl of Optilyse C Solution (Beckman Coulter, High Wycombe, UK) added to all tubes which then were vortexed and left for 10 min. The final step was to add 500 μl of PBS to all tubes, followed by 100 μl of counting beads. The prepared tubes were left overnight at 4°C then analyzed using a Beckman Coulter FC500 flow cytometer. The bead count number was inserted into the protocol and the samples run on a low/medium speed. Gating criteria were set and data analyzed using WinMDI 2.8 software.

### Western blotting

Snap-frozen myometrial biopsies (0.5 g wet weight) and choriodecidua (0.5 g wet weight) were homogenized on ice in homogenization buffer (pH 7.8) consisting of sucrose (320 mM), Tris (10 mM), KCl (50 mM), EDTA (1 mM), Igepal 0.5% and 1/500 and 1/100 respective dilutions of protease inhibitor cocktail and 1/100 dilutions of phosphatase inhibitor cocktail (Sigma-Aldrich, Poole, UK). The resulting homogenate was then sonicated for 15 min followed by shearing through a 21 g needle. Samples were then centrifuged (1500 g at 4°C for 10 min), the resulting supernatant collected and centrifuged again (14,000 g at 4°C for 1 h) then frozen at −80°C for western blotting. Preparations of CBMC, PLBMC, and MBMC (10 million cells/ml of each sample) were prepared for western blotting using two rounds of a freeze-thaw cycle then passing through a 21 g needle as for myometrium and choriodecidua.

For SDS-PAGE, lysates of choriodecidua, myometrium, and leukocytes were diluted with Laemmli buffer (BioRad, Hemel Hempstead, UK) containing 5% β-mercaptoethanol. Approximately 10 μl of each sample were loaded and electrophoresed for 50–60 min at 40 mA. The wet transfer onto nitrocellulose membranes was carried out using a BioRad Miniprotean III system for 2 h at 100 V at 4°C. After electroblotting and confirmation of equal loading and transfer of proteins with Ponceau S red solution, (Sigma-Alrich, Poole, UK), blots were washed with distilled water three times then blocked in 5% Marvel milk protein in TBS for 1 h followed by probing with primary antibodies against TLR4, P2X_7_, prolactin receptor (Abcam, Cambridge, UK) and the P4 receptor (Abcam, Cambridge, UK). Antibodies were prepared in 3% Marvel milk/TBS, added to each blot at the appropriate dilution and membranes left shaking gently overnight at 4°C then washed the next day to remove any unbound antibody. Alkaline phosphatase-conjugated secondary antibody (diluted in 3% Marvel milk protein/TBS) was then added to immunoblots for 2 h at room temperature. Protein bands were detected using the non-isotopic chemiluminescent detection system Immuno-star-Alkaline Phosphatase (AP) kit (BioRad, Hemel Hempstead, UK), viewed using the Chemi Doc (version 4.2.1) imaging system. Blots were then stripped using Reblot Plus (Millipore) for 12 min and re-probed to measure β-actin expression as a loading control where it was used for qualitative purposes only.

### Immunofluorescence

Culture media from mononuclear cell preparations was removed, cells washed with PBS followed by fixation in a 1:1 mix of ice-cold acetone:methanol mixture for 10 min. Following three further washes with PBS, 20% goat serum was added to each well and plates incubated for 30 min at room temperature on a shaking platform. Then 200 μl of corresponding antibody prepared in 20% goat serum were added to corresponding wells after aspiration of blocking solution and plates incubated overnight at 4°C with shaking. On the next day, primary antibodies were removed and each well-washed with 1 ml of PBS for 10 min, three times. Secondary fluorescein isothiocyanate (FITC)-labeled antibodies (Abcam, Cambridge, UK) were made in blocking solution with 250 μl of secondary antibody added to each well. The plates were then covered in foil and incubated with shaking for 30 min at room temperature. Secondary antibodies were then aspirated and the wells washed with 1 ml of PBS for 10 min, three times. Finally, 1 ml of PBS was added to each plate which was then examined in a dark room with a Ziess Axiovert 25 fluorescence microscope and photographed (Olympus DP70 digital camera) and Cell F software. Addition of control IgG instead of the test primary antibody enabled specific from non-specific staining to be identified and was included in all immunofluorescence/flow cytometry experiments.

For indirect immunofluorescence staining for surface and transmembrane proteins by flow cytometry, between 100 and 500 μl of cell suspension containing 5 × 10^6^ cell/ml of purified mononuclear cells were aliquoted into labeled test tubes as required including a tube with neat cell suspension (control), another with primary antibody (to assess antibody concentration) and a tube with secondary antibody alone (negative control). Following addition of the appropriate primary antibody, tubes were incubated at room temperature for 60 min followed by washing of cells by adding 1 ml of PBS/BSA then centrifuging at 300 g for 5 min. The resulting supernatant was carefully removed an appropriate dilutions of the secondary antibody added to corresponding tubes and mixed well following incubation at room temperature for 30 min. After washing with PBS/BSA, solutions were centrifuged at 300 g for 5 min, the supernatants discarded and cells fixed in 1 ml of 4% paraformaldehyde for 10–15 min at 4°C. Cells were washed again with PBS/BSA buffer, and centrifuged at 300 g for 5 min then resuspended in 500 μl of PBS/BSA then data was acquired by flow cytometry.

### Cytokine release from intact membranes using transwells

Full-thickness gestational membranes comprising amnion, chorion and decidua were dissected intact under sterile laboratory conditions. The membranes were examined to ensure that the amnion and choriodecidua remained attached to one another. Culture medium consisted of RPMI supplemented with 1% (v/v) heat inactivated-fetal bovine serum, 100 units penicillin/ml, and 100 μg streptomycin/ml (Zaga et al., 2004; Thiex et al., 2009, studies, 2010). Full-thickness gestational membranes were cultured using a Transwell two-chamber tissue culture. The membranes were cut into approximately 2 × 2 cm^2^ pieces, affixed by elastic latex bands onto ethylene oxide sterilized Transwell frames (with the synthetic membrane removed). The outer 2 cm of the gestational membranes was excluded from experimentation. Membranes were studied intact with drugs added directly to the upper chamber with choriodecidua uppermost. Experiments were also conducted with the amnion uppermost. Fetal membranes in the Transwell assembly were maintained over 24 h then challenged as described for cells with LPS followed by BzATP, the latter being added 30 min before the end of the stimulation protocol.

### Statistical analysis

Results are expressed as mean ± SD. Quantitative data generated by ELISA were analyzed with Prism V6 software (Graphpad). Statistical analysis was carried out using one-way analysis of variance with *post-hoc* Bonferroni correction, the Kruskall-Wallis test for non-parametric data and paired *t*-test for comparison between L and NL groups. A value of *P* < 0.05 was considered to indicate statistical significance.

## Results

### Characterization of cells in whole and purified cord blood

**Figure 1:**
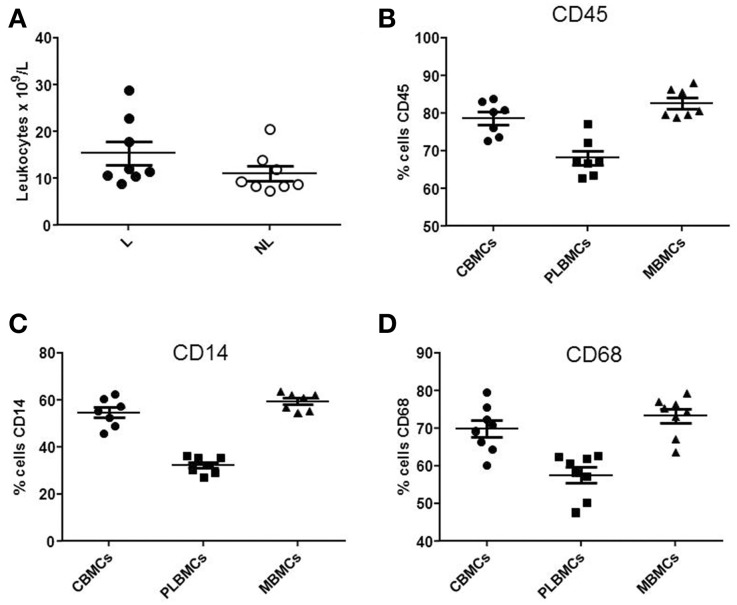
**Leukocyte populations in fetal and maternal blood. (A)** Differences between cell marker expression in preparations of MBMCs, CBMCs, and PLBMCs using flow cytometry (*n* = 6–10 in each group). Leukocyte count in whole cord venous blood of L group and NL group was significantly different between the two the groups (*P* < 0.05). **(B)** Scatter plot showing the percentage of CBMCs, PLBMCs, and MBMCs expressing **(B)** CD45, **(C)** CD14, and **(D)** CD68, determined by flow cytometry.

**Figure 2:**
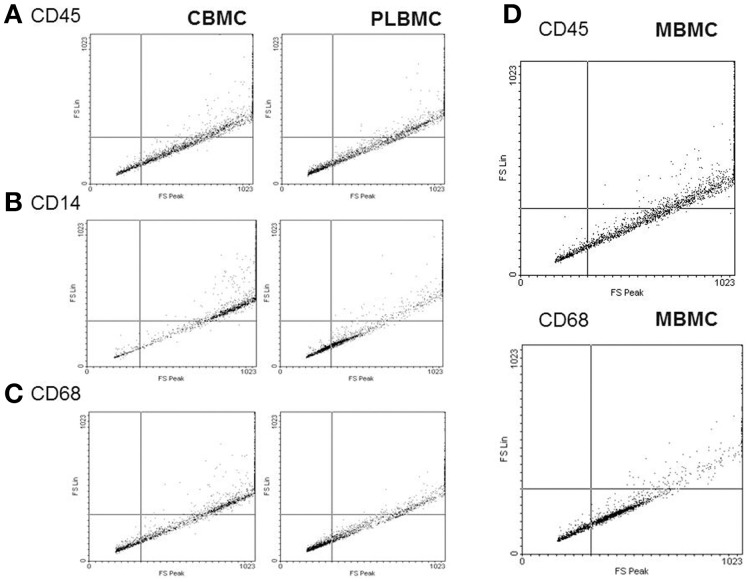
**Dot plot of leukocyte markers in isolated cells**. Cells were prepared for flow cytometry then analyzed using fluorescence leukocyte markers CD45 **(A)**, CD14 **(B)** and CD68 **(C)**. Levels of CD14 **(B)** were lowest in PLBMCs, CBMCs, and MBMCs. **(D)** Dot plot for MBMCs.

Whole blood from the umbilical vein and intervillous space of the placenta L (*n* = 6–10) and NL (*n* = 6–10) was analyzed by flow cytometry. The total leukocyte count (Figure 1A) was in the range 10.64–28.82 × 10^9^/L in L group compared with 7.19–20.45 × 10^9^/L in NL group (*P* < 0.05). Figure 1 shows that CBMCs and MBMCs expressed higher amounts of the pan-leukocyte marker CD45 (Figure 1B), the monocyte markers CD14 (Figure 1C) and CD68 (Figure 1D) than PLBMCs (*P* < 0.05) while proportions between CBMCs and MBMCs were similar (*P* > 0.05). Flow cytometry data based on scatter and fluorescence for CD45, CD14, and CD68 is shown in Figure 2.

### TLR4 and P2X_7_ expression in mononuclear cells, myometrium, and choriodecidua

**Figure 3:**
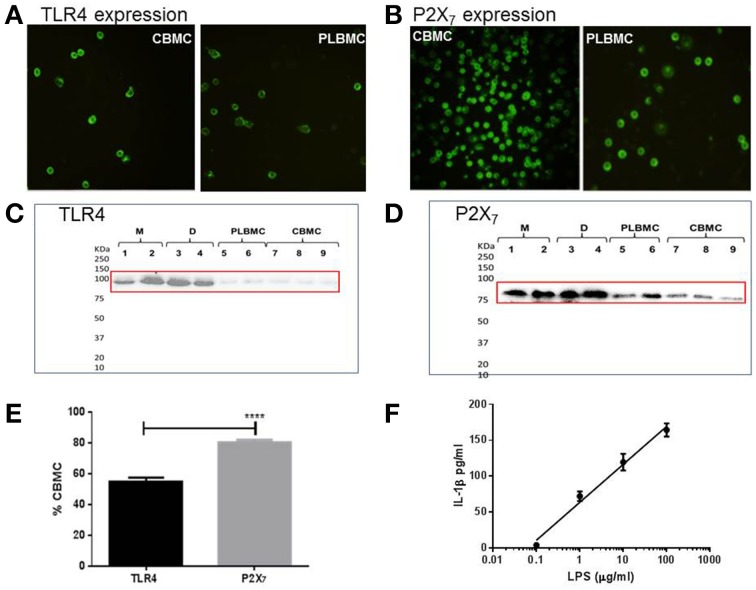
**Expression of TLR4 and P2X_7_ proteins in gestational tissues. (A)** CBMCs and PLBMCs express TLR4 (anti-TLR4 antibody at 1:200); **(B)** CBMCs and PLBMCs express the P2X_7_ receptor (anti-P2X_7_ antibody at 1:200). Immunofluorescence was detected using fluorescence-tagged secondary antibodies following fixation and incubation with a primary antibody. No background fluorescence was visible in cells that had been preincubated with control IgG instead of the primary antibody **(C)** Representative western blots obtained using lysates prepared from myometrium (M; *n* = 5), choriodecidua (D; *n* = 5), PLBMCs (*n* = 7), and CBMCs (*n* = 7) identifies an 85 kDa using anti-TLR4 antibody and **(D)** an anti-P2X_7_ antibody that recognizes 74–78 kDa protein. **(E)** Using flow cytometry, a higher percentage of CBMCs (*n* = 7) expressed the P2X_7_ receptor (80.2%) compared with TLR4 where 55.0% were positive (*n* = 7). **(F)** Concentration-dependence of IL-1β release in response to LPS from CBMCs.

CBMCs (*n* = 8) and PLBMCs (*n* = 7) expressed both TLR4 (Figure 3A) and P2X_7_ receptor (Figure 3B) proteins as detected by immunofluorescence along with MBMCs (data not shown). Western blotting further corroborated the presence of TLR4 immunoreactivity in PLBMCs (*n* = 7) and CBMCs (*n* = 7) as well as expression in myometrium (*n* = 5) and choriodecidua (*n* = 5) of NL (Figure 3C) and L samples (Data not shown). P2X_7_ receptor expression was clearly detectable with bands of high intensity of the expected molecular weight (74–78 kDa) in myometrium, choriodecidua, PLBMCs and CBMCs (Figure 3D). Flow cytometry showed that some 55.0% of CBMCs express TLR4 (*n* = 7) while more than 80% of these cells express P2X_7_ (*n* = 7; Figure 3E). In PLBMCs, the results were comparable to those in CBMCs where 47.8% of cells express TLR4 while a similarly higher number (70.8%) of cells express P2X_7_. CBMCs responded to LPS (0.1–1000 μgml^−1^) by producing concentration-dependent release of mature IL-1β detectable in cell supernatant (*n* = 4; Figure 3F) and informed our dose of LPS in the study.

**Figure 4:**
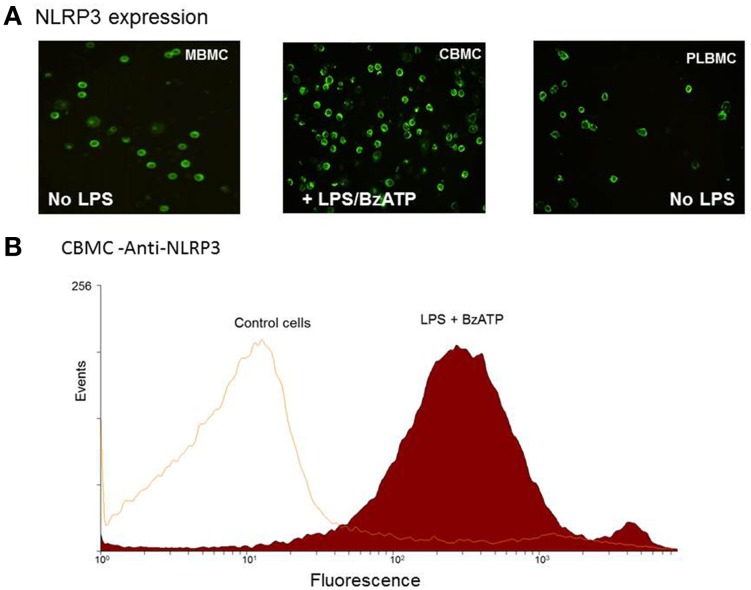
**Expression of NLRP3 in MBMCs, PLBMCs, and CBMCs. (A)** Expression of P2X_7_ was detected by immunofluorescence in MBMCs (LH panel), CBMCs (middle panel) and PLBMCs (right hand panel) with CBMCs requiring prior incubation with LPS + BzATP before NLRP3 was detectable. **(B)** Flow cytometry demonstrated that costimulation with LPS + BzATP resulted in 92.4% of CBMCs showing NLRP3 expression as seen in the single parameter histogram.

In order to examine a role for NLRP3 in the production of IL-1β, MBMCs (*n* = 5), CBMCs (*n* = 5) and PLBMCs (*n* = 5) from the NL group were tested unstimulated and following stimulation with 1 μg/ml LPS. In contrast to MBMCs (Figure 4A, left hand panel) and PLBMCs (Figure 4A; right hand panel), unstimulated CBMCs did not express NLRP3 but it was induced following stimulation with LPS + BzATP (Figure 4B, middle panel). Flow cytometry (*n* = 5) confirmed positive immunofluorescence for NLRP3 in CBMCs (92.4%) following induction with LPS + BzATP (Figure 4B) compared with a lack of expression in control, unstimulated cells.

### The role of TLR4 and P2X_7_ in triggering of IL-1β secretion in CBMC

**Figure 5:**
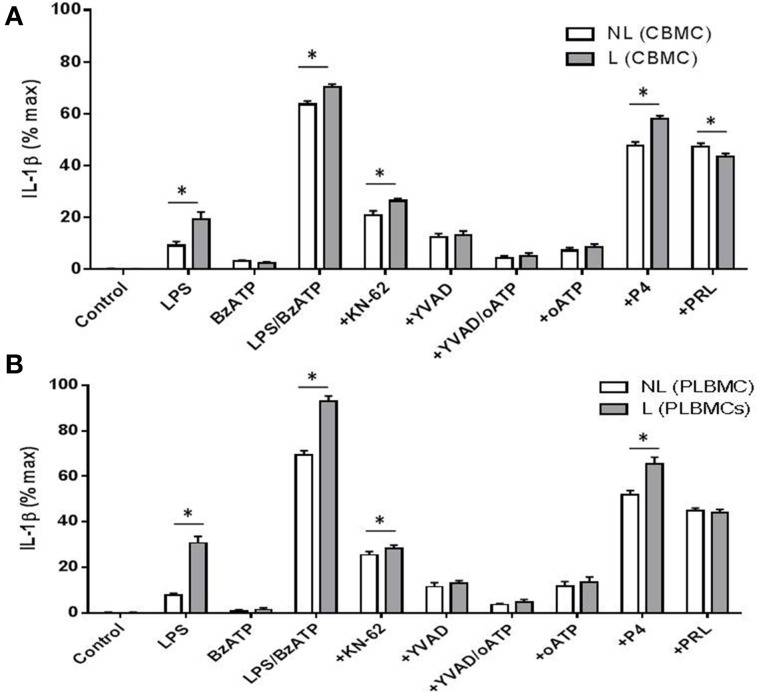
**CBMCS and PLBMCs from laboring (L) and non-laboring (NL) women incubated with LPS and BzATP leads to increased IL-1β release**. Costimulation with LPS and BzATP led to increased release of IL-1β than either LPS or BzATP alone from **(A)** CBMCs and **(B)** PLBMCs. Cells from laboring women produced significantly greater amounts of IL-1β. IL-1β release was inhibited following preincubation with the P2X_7_ antagonists KN-62, oATP and the caspase -1 inhibitor YVAD-CHO following costimulation with LPS and BzATP as indicated on graphs by a “+” prefix before treatment. A combination of YVAD and oATP led to greater inhibition of IL-1β than either inhibitors alone, Both LPS and BzATP_evoked IL-1β release which was inhibited by P4 and prolactin in **(A)** CBMCs and **(B)** PLBMCs. (^*^P < 0.05).

Stimulation of TLR4 in CBMCs (*n* = 8) and PLBMCs (*n* = 8) with LPS and LPS + BzATP triggered the production of IL-1β in cell supernatant in both L and NL groups and was significantly higher in the L group (*P* < 0.05) in both preparations (Figures 5A,B). The P2X_7_ agonist BzATP alone had no effect on IL-1β release from either CBMCs or PLBMCs compared with control, unstimulated cells (Figures 5A,B; *n* = 6–10 in cases). To evaluate further, the individual role of the P2X_7_ receptor in IL-1β release, preincubation with the P2X_7_ antagonists 10 μM KN-62 (Warren et al., 2008) alone to cells stimulated with LPS + BzATP inhibited IL-1β release (*P* < 0.05) by over 60% (Figures 5A,B). IL-1β release in both CBMCs and PLBMCs was also significantly attenuated by the caspase-1 inhibitor YVAD-CHO to a similar extent between the L and NL groups in CBMCs and PLBMCs on LPS + BzATP stimulation. A combination of YVAD-CHO and oATP produced a greater inhibition of IL-1β release than either agent applied alone in cells of both L and NL groups (Figures 5A,B).

In CBMCs and PLBMCs preincubated with P4 (1 μM) and stimulated with LPS + BzATP, a significant reduction in the release of IL-1β was noted (Figures 5A,B) which was greater in PLBMCs (Figure 5B) and in L (*n* = 6) relative to the NL group (*n* = 6; *P* < 0.05). The effects of P4 were dose-dependent (10^−8^–10^−5^ M, data not shown). Prolactin (1 μM), also in cells prestimulated with LPS + BzATP, inhibited IL-1β release from CBMCs (Figure 5A) and PLBMCs (Figure 5B) in L and NL women with no differences noted between L and NL samples. Repeating the experiment in the presence of QNZ (200 nM) also demonstrated significant inhibition in cells from L (*n* = 4) and NL women (*n* = 4; data not shown).

### The role of TLR4 and P2X_7_ in triggering of IL-1β secretion in MBMCS

**Figure 6:**
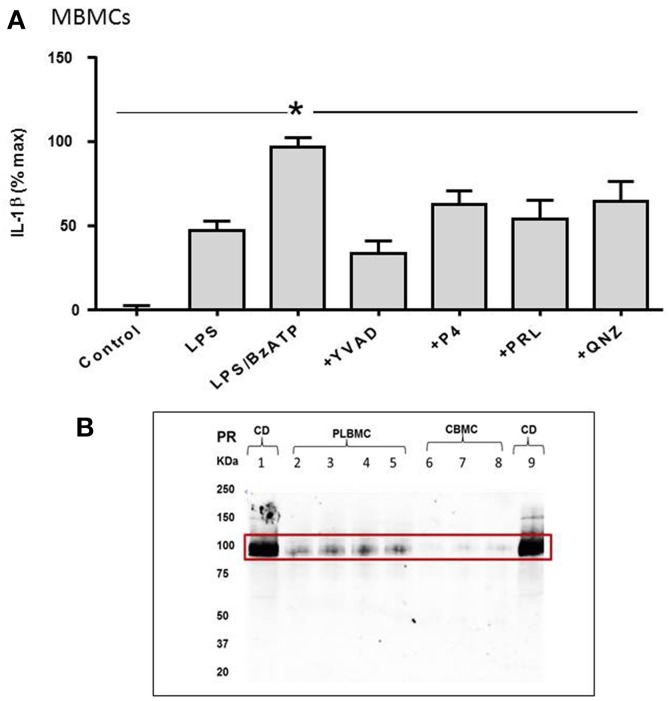
**IL-1β release from maternal blood mononuclear cells (MBMCs). (A)** Coincubation with LPS and BzATP elicited greater production of IL-1β from MBMCs. P4, prolactin, the NFkB inhibitor QNZ and the caspase-1 inhibitor YVAD-CHO all inhibited IL-1β release. **(B)** Western blotting showed positive expression for the P4 receptor in choriodecidua (CD; *n* = 6), some expression in PLBMCs (*n* = 6) but undetectable levels in CBMCs (*n* = 6). ^*^P < 0.05 when compared using Dunnett's test.

MBMCs displayed a similar response to LPS and BzATP to that observed in CBMCs and PLBMCs. As is evident in Figure 6A, IL-1β release provoked in MBMCs was consistent with that noted in CBMCs and PLBMCs (Figure 5). LPS alone increased the IL-1β released from baseline in the control group which was doubled by LPS + BzATP but reduced in the presence of YVAD-CHO in cells preincubated with LPS + BzATP (Figure 6A). The effect of QNZ was less than that of YVAD-CHO (Figure 6A). Interestingly, both P4 and prolactin partially inhibited the LPS + BzATP-mediated increase in IL-1β (Figure 6A).

Western blotting provided evidence for expression of the P4 receptor in choriodecidua and PLBMCs but not CBMCs (Figure 6B; *n* = 6 for all) while prolactin receptor protein was undetectable in CBMCs in contrast to myometrium, choriodecidua and a faint signal in PLBMCs (*n* = 6; data not shown).

**Figure 7:**
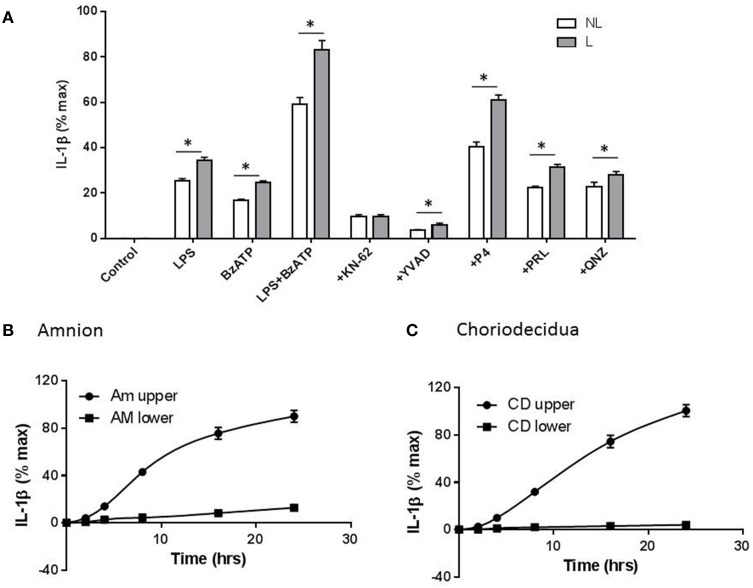
**Gestational membranes produce IL-1β**. **(A)** Intact membranes assembled in transwells stimulated with LPS, BzATP, and LPS+ BzATP led to increased IL-1β release which was highest in the latter and from laboring samples. KN-62, YVAD-CHO, progesterone (P4), prolactin (PRL), and (QNZ) all caused a reduction in IL-1β release in cells stimulated with LPS + BzATP combined. **(B)** Using intact membranes and sampling media on both sides (upper) and (lower) of the transwell, amnion produced IL-1β only in the upper compartment **(C)** when choriodecidua was uppermost and upper and lower chambers tested for IL-1β production, higher levels of the cytokine were produced from the upper chamber and were higher than those produced when amnion was uppermost (*n* = 6; *P* < 0.05). IL-1β amounts are expressed per/cm^2^. ^*^P < 0.05 comparing each treatment between NL and L groups.

In order to determine whether fetal membranes play a role in the release of IL-1β, intact membranes in transwells were incubated with test agents applied to the choriodecidual surface and IL-1β in the upper chamber measured by ELISA. IL-1β release was highest in membranes stimulated with LPS + BzATP. In the presence of the latter, 10 μM KN-62 (*n* = 5), YVAD-CHO (*n* = 5), 10^−5^ M P4 (*n* = 8), 1 μM prolactin (*n* = 6) and 200 nM QNZ (*n* = 6) all caused inhibition of IL-1β secretion consistent with effects noted in CBMCs, PLBMCs and MBMCs with the largest degree of inhibition seen with KN-62 and YVAD (Figure 7A). It was also apparent that fetal membranes from laboring women produced higher amounts of IL-1β than the NL group for all treatments except with KN-62 inhibition (Figure 7A). Interestingly inhibition with prolactin was greater in fetal membranes than isolated mononuclear cells. When LPS + BzATP were coapplied to the upper chamber with amnion uppermost, IL-1β was produced in the upper chamber with only low amounts detected in supernatant sampled from the lower chamber over 24 h (*P* < 0.05; *n* = 6; Figure 7B). Similar effects were observed with choriodecidua uppermost (Figure 7C; *n* = 6).

## Discussion

The P2X_7_ receptor provides an intriguing link in transducing the signals elicited by bacterial endotoxin via the TLR4 receptor and the secretion of the cytokine IL-1β (Colomar et al., 2003; Kahlenberg et al., 2005; Mariathasan et al., 2006), previously shown to be raised in PTB (Romero et al., 1989, 1992). In relation to pregnancy, the function and physiology of P2X_7_ receptors in gestational tissues and immune cells at the fetomaternal interface are not known. We have previously reported on the role of the P2X_7_ receptors in CBMCs (Warren et al., 2008) and herein confirm P2X_7_ receptor expression in PLBMCs, MBMCs, myometrium, and choriodecidua. Our results also verify protein expression of TLR4 in myometrium (Dallot et al., 2005; Youssef et al., 2009) and choriodecidua (Krikun et al., 2007) as well as CBMCs and PLBMCs as reported for myeloid cells in pregnancy (Youssef et al., 2009).

A key finding of our study is that the largest increase in release of IL-1β from CBMCs, PLBMCs, and MBMCs, is evoked by costimulation with LPS and the P2X_7_ agonist, BzATP, and is maximally inhibited in the presence of the caspase-1 inhibitor YVAD-CHO implicating inflammasomes in release of IL-1β. Indeed, our finding of NLRP3 inflammasome expression in PLBMC and MBMCs in the absence of stimulation with LPS and BzATP implies constitutive expression of this protein. In contrast, CBMCs did not express the NLRP3 inflammasome unless primed with LPS or a combination of LPS and BzATP. We also observed constitutive expression of ASC protein in CBMCs, PLBMCs, and MBMCs (data not shown). A possible explanation for these observations is that the NLRP3 inflammasome is already functional in PLBMCs since the latter isolated from the intervillous space derive largely from the maternal circulation and are likely to be a mix of cell types including maternal leukocytes and fetal macrophages (Hofbauer cells) compared with relatively immature fetal monocytes cells that comprise cord blood; this also explains the lower percentage of CD14^+^ and CD68^+^ cells in the PLBMC preparation.

Recently, it was reported that the ability of CBMCs to secrete IL-1β is dependent on a high CD14-expressing monocyte subset producing pro-1L-1β but characterized by impaired release of IL-1β attributed to low expression of NLRP3; P2X_7_ and pro-caspase-1 expression were comparable to adult levels (Sharma et al., 2015). Sharma *et al.*, also found that a low CD14-expressing monocyte subset, abundant prior to 33 weeks of gestation, was incapable of producing IL-1β following costimulation with LPS and ATP (Sharma et al., 2015). They were also able to show that high CD14-expressing monocyte subset obtained at term required stimulation with LPS before NLRP3 could be detected (Sharma et al., 2015) and agrees with our observations that CBMCs required priming by LPS for translation of NLRP3.

Leukocyte trafficking to the uterus is increased with parturition (Thomson et al., 1999; Osman et al., 2003) which led to us further investigate the P2X_7_ pathway in gestational membranes based on the fact that the receptor is expressed abundantly in immune cells. Stimulation of IL-1β release was tested using intact disks of fetal membranes *ex vivo* using the two-chamber transwell model (Zaga et al., 2004). Our finding that IL-1β release from intact fetal membranes cases is mediated by the P2X_7_ receptor pathway reflects data obtained from isolated CBMCs and PLBMCs. In a recent study using fetal membranes and myometrium obtained prior to and after the onset of labor, a potential role for the inflammasome was inferred from increased gene transcription of caspase-1 (Lappas, 2014). Moreover, using explants of membranes and myometria, LPS and ATP stimulated IL-1β production which was further enhanced with nigericin and inhibited using KN-62 (Lappas, 2014). Fetal membranes have also been shown to express all ten human TLR subtypes in addition to NLRP1, NLP3, ASC, and caspase-1 (Hoang et al., 2014). In the latter study, it was also shown that active IL-1β levels were higher from fetal membranes after stimulation with PAMPs including LPS with IL-1β only noted on stimulation with TLR-2, -4 and, -5 ligands (Hoang et al., 2014). In our study, consistent with previous findings (Zaga et al., 2004), stimulation of choriodecidual membranes with LPS produced elevated IL-1β production in response to LPS compared with amniotic membranes suggesting that the choriodecidual side of gestational membranes appears more responsive to TLR4 and P2X_7_ stimuli compared with the amnion. Others have shown that treating the choriodecidual side of the membrane leads to production of IL-6, IL-10, IL-8, and TNF-α levels in the same compartment while only TNF-α and IL-6 levels were found in the lower compartment (Thiex et al., 2009); levels of IL-1β were not statistically significant irrespective of LPS application to either amniotic or choriodecidual aspects (Thiex et al., 2009). In other studies and in agreement with our data, IL-1β release was higher from the choriodecidual side (Pineda-Torres et al., 2014). A greater range and amount of cytokines produced by choriodecidua is not surprising, given that this barrier likely mounts a stronger response in providing protection for the fetus from maternal infection. A similar effect is seen for TLR4 receptors that show higher expression in trophoblast membranes facing maternal blood in the intervillous space (Beijar et al., 2006).

Based on reduced levels of IL-1β release in leukocytes and choriodecidual membranes following incubation with P4 (Pineda-Torres et al., 2014), an immunomodulatory role for this steroid appears justified. As fetal and neonatal CBMCs lack nuclear P4 receptors, postulated mechanisms for the anti-inflammatory effects of P4 include actions at membrane P4 receptors (Giannoni et al., 2011), effects on cAMP cascades (Schwartz et al., 2009) or by inhibition of the NF-κB pathway (Srivastava and Anderson, 2007; Giannoni et al., 2011). Of course, P4 may also exert anti-inflammatory effects through glucocorticoid receptors expressed in cord blood (Imamura et al., 2011). It is unlikely that P4's effects are via inhibition of current through P2X_7_ receptors given the lack of effect observed with application of the steroid (Cario-Toumaniantz et al., 1998). Similar uncertainty exists around the anti-inflammatory effects we observed with prolactin which attenuated the amount of IL-1β produced by CBMCs, PLBCs, MBMCs, and fetal membranes costimulated with LPS and BzATP. A recent finding also noted inhibition of IL-1β in fetal membranes by prolactin but over a much longer time course of 32 h (Zaga-Clavellina et al., 2014). Addition of LPS activated the synthesis of prolactin receptor mRNA and prolactin mRNA in human monocytes *in vitro* up to 300- and 130-fold, respectively (Lopez-Rincon et al., 2013) and is interesting in the context that human decidua produces large amounts of prolactin throughout pregnancy (Rosenberg et al., 1980), that like P4, may play a role in local host-defense responses.

Extracellular levels of ATP, the natural ligand for the P2X_7_ receptor, are tightly regulated *in vivo* but achieve amounts required for activation of the P2X_7_ receptor in the presence of necrosis or tissue injury. This links the P2X_7_ receptor to events in the pregnant uterus where levels of extracellular ATP levels may become elevated through localized tissue damage caused by the powerful contractions of the myometrium, resulting in DAMP-dependent inflammasome activation.

Inflammatory mechanisms remain a crucial area of research in understanding pregnancy disorders particularly the mechanisms deployed by harmful microbes to breach physical barriers but also maternal and fetal defenses that are designed to resist infection. We saw no obvious differences amongst the various preparations used in this investigation except that higher IL-1β levels were secreted in cells and tissues isolated from women in labor, suggesting that the TLR4-P2X_7_-IL-1β axis is a common pathway within the uterus and fetal membranes. It has been suggested that myometrial contractility may result from COX-2 mediated prostaglandin production induced by inflammatory stimuli that include IL-1β (Rauk and Chiao, 2000). Coupled with this, it was demonstrated that overnight incubation of myometrial strips with IL-1β led to significant release of prostaglandins (Oger et al., 2002). Alternatively, production of IL-1β via P2X_7_ receptor activation in immune cells could also drive myometrial contractility. Indeed, expression of the rat myometrial P2X_7_ receptor increases prior to labor and in an animal model of PTB, P2X_7_ mRNA and protein increased closer to term indicating a distinct activation of inflammatory pathways (Urabe et al., 2009; Miyoshi et al., 2010). This group also reported an ATP-activated conductance, presumed to be P2X_7_, in rat myometrium (Miyoshi et al., 2012).

In conclusion, a role for TLR4 and P2X_7_ receptors in gestational tissues and cells is suggested. Both were expressed in leukocytes isolated from cord venous blood but also from maternal blood in the intervillous space. The expression of P2X_7_ in human myometrium and choriodecidua provides support for a P2X_7_ pathway of potential importance in paracrine interactions between maternal and fetal systems involving smooth muscle as well as immune cells but also fetal membranes. However, some aspects of fetal immunity appear to be immature as evidenced by the priming required for NLRP3 expression in CBMCs but not for PLBMCs and MBMCs. Release of the cytokine IL-1β via inflammasome activation downstream of P2X_7_ activation may enable direct signaling between the amnion and choriodecidua thereby triggering prostaglandin-mediated myometrial contractions but may also constitute a first-line of immune defense to microbes reaching the amniotic cavity by amniotic production of IL-1β. Differences were observed with higher levels of IL-1β secreted in cells and tissues derived from laboring compared with non-laboring samples. The modulation of IL-1β release by P4, prolactin and an NFkB inhibitor in mononuclear cells raises the possibility of targeting the P2X_7_ receptor pathway to limit inflammation and is also a novel mechanism through which P4 may help prevent PTB (da Fonseca et al., 2003). Currently, there is little direct evidence available for exaggerated inflammasome activity as a factor in causing PTB yet clues point to a potential association. Interestingly, a recent study examining polymorphisms in genes associated with inflammation observed an altered frequency for the gene CIASI (NLRP3) indicating a potential genetic predisposition to PTB (Jaffe et al., 2013). Investigations into studies of the chain of events involved in inflammasome activation in cases of sPTB compared with normal parturition may yield further insight into mechanisms and new targets that enable prediction of PTB. Specifically, a need to better understand the NLRP3-linked signal transduction pathways activated by bacterial toxins implicated in sPTB may help unravel the complexity of inflammatory mechanisms implicated in fetal demise as a result of being born too early.

### Conflict of interest statement

The authors declare that the research was conducted in the absence of any commercial or financial relationships that could be construed as a potential conflict of interest.
